# Transcriptome Analysis Reveals the Mechanism of Quinoa Polysaccharides Inhibiting 3T3-L1 Preadipocyte Proliferation

**DOI:** 10.3390/foods13152311

**Published:** 2024-07-23

**Authors:** Cong Teng, Shengyuan Guo, Ying Li, Guixing Ren

**Affiliations:** 1 Institute of Agro-Product Processing, Jiangsu Academy of Agricultural Sciences, Nanjing 210014, China; tengcong95@163.com; 2 College of Food and Bioengineering, Chengdu University, Chengdu 610106, China; 3 College of Life Science, Shanxi University, Taiyuan 030006, China

**Keywords:** transcriptome, quinoa, polysaccharides, 3T3-L1, cell proliferation

## Abstract

Quinoa is a highly nutritious and biologically active crop. Prior studies have demonstrated that quinoa polysaccharides exhibit anti-obesity activity. This investigation confirmed that quinoa polysaccharides have the ability to inhibit the growth of 3T3-L1 preadipocytes. The objective of transcriptome research was to investigate the mechanism of quinoa water-extracted polysaccharides and quinoa alkaline-extracted polysaccharides that hinder the growth of 3T3-L1 preadipocytes. There were 2194 genes that showed differential expression between untreated cells and those treated with high concentrations of quinoa water-extracted polysaccharides (QWPHs). There were 1774 genes that showed differential expression between untreated cells and those treated with high concentrations of quinoa alkaline-extracted polysaccharides (QAPHs). Through gene ontology and KEGG pathway analysis, 20 characteristic pathways are found significantly enriched between the untreated group and the QAPH and QWPH groups. These pathways include the NOD-like receptor, Hepatitis C, and the PI3K-Akt signaling pathway. Atp13A4 and Gbgt1 have been identified as genes that are upregulated and downregulated in both the untreated group and the QWPH group, as well as in the untreated group and the QAPH group. These findings establish a theoretical foundation for exploring quinoa polysaccharides as an anti-obesity agent.

## 1. Introduction

Quinoa (*Chenopodium quinoa Willd.*) is a kind of pseudocereal native to the Andes of South America, and contains protein, polysaccharides, unsaturated fatty acids, minerals, and saponins [1]. Recent studies have shown that quinoa has antioxidant [2], immunomodulatory [3], and anti-cancer activities in vitro and in vivo [4]. Polysaccharide is an important class of biological macromolecules, which participate in and mediate the regulation of various cell life phenomena. Many non-starch polysaccharides exhibit anti-obesity effects [5]. Quinoa polysaccharides, as important active substances in quinoa, have exhibited multiple biological activities [6]. 

Obesity is an independent metabolic disorder that brings about a more complex set of health problems, including type II diabetes, cancer, hypertension, and other cardiovascular diseases. Hyperplastic adipogenesis and adipocyte hypertrophy are important causes of obesity [7]. Obesity caused by the excessive accumulation of adipose tissue is one notable health issue [8]. The increase in obesity cells is caused by cell division and proliferation. Some polysaccharides are used to control the proliferation and differentiation of fat cells, thereby achieving anti-obesity effects [9]. 3T3-L1 preadipocytes are widely used in the study of anti-obesity drug application because they can differentiate mature fat cells in vitro [10]. However, their strong proliferation capacity seems to be ignored. The proliferation of preadipocytes is associated with the formation of adipocytes, and obesity can be treated by inhibiting the proliferation of preadipocytes [11]. Previous research reported that quinoa polysaccharide significantly inhibited 3T3-L1 preadipocyte differentiation to achieve an anti-obesity effect [12]. Elucidating the antiproliferative mechanism of quinoa polysaccharides on 3T3-L1 preadipocytes can clarify the signaling pathway, which provides a basis for quinoa anti-obesity functional food. In addition, anti-obesity drugs have some side effects. Quinoa polysaccharides are safer compared to anti-obesity drugs. The mechanism that quinoa polysaccharides use for inhibiting the proliferation of 3T3-L1 preadipocytes deserves an in-depth study. 

There were several prior studies conducted to analyze the transcriptome sequencing of adipocyte and tumor cells in order to identify functional genes. In Ying’s study, transcriptome analysis was used to reveal that Neurotrophin, VEGF, and the P53 signaling pathway were identified and related to phycocyanin’s inhibitory effects on SKOV-3 cells [13]. Smilax glabra Roxb (SGF) can induce the expression of genes involved in triglyceride storage, fatty acid β-oxidation, and mitochondrial biogenesis [14]. Other related transcriptional changes are related to the AMPK/PGC-1α signaling and inflammation and PI3K/AKT signaling pathways.

This study was conducted to investigate the impact of quinoa polysaccharides on the proliferation of 3T3-L1 preadipocytes. High-throughput sequencing technology was used to analyze transcripts of different concentrations of quinoa water-extracted polysaccharides (QWPs), as well as quinoa alkaline-extracted polysaccharide (QAP)-treated and untreated groups at the transcriptome level. The mechanism used by quinoa polysaccharides for regulating 3T3-L1 preadipocyte proliferation was elaborated. It is helpful to provides a basis for the development of a quinoa anti-obesity agent. 

## 2. Materials and Methods

### 2.1. Sample and Reagents

Quinoa was obtained from the Institute of Crop Sciences, the Chinese Academy of Agricultural Sciences. Dulbecco’s modified Eagle’s medium (DMEM), Penicillin–Streptomycin 10,000 U/mL, fetal bovine serum (FBS), Trypsin–EDTA Solution, and α-amylase were purchased from Sigma-Aldrich (St. Louis, MO, USA). Ethanol, N-hexane, sodium hydroxide, HCl, chloroform, and N-butanol were of analytical grade, and they were purchased from Beijing Chemistry Industry Group Co., Ltd. (Beijing, China).

### 2.2. Quiona Polysaccharide Preparation

Methods of polysaccharide extraction and purification were used according to previous research [2,12]. Quinoa seeds were milled and sieved over 60 mesh, then soaked in N-hexane for 6 h to remove lipids and pigments. The QWP extraction method was employed as follows. We skimmed quinoa powder and extracted it twice at 1:10 with distilled water (90 °C, 4 h). The QAP extraction method was employed as follows. Sodium hydroxide (1.25 mol/L) was added to the pre-treated skimmed quinoa powder at 1:10 (*w*/*v*) and extracted twice for 3 h. The extract was mediumed to pH 7.0 and centrifuged (4000× *g*, 20 min, 4 °C) using a centrifuge (H1850R, Cence, Changsha, China). The supernatant was combined with ethanol for 12 h at a ratio of 1:5 at 4 °C, then centrifuged (5000× *g*, 15 min, 4 °C) to collect sediment. The precipitate was then deproteinized by using the Sevage method. To remove starch and perform dialysis, the fraction was fully treated with α-amylase, and dialysis was performed. The QAP and QWP fractions were obtained by freeze-drying using a freeze-drier (LGJ-12, Songyuan, China) from Beijing Songyuan Huaxing Technology Development Co., Ltd. The QAP and QWP fractions were separated and purified by an ion-exchange column and a sephadex column. In brief, the eluting components were dialyzed and freeze-dried to obtain a column chromatography separation of DEAE Sepharose Fast Flow. Further purification was carried out using the Sephacryl S-300 system to obtain a new component.

### 2.3. Measurement of Anti-Proliferation Activity

3T3-L1 preadipocytes were purchased from the Chinese Academy of Sciences Shanghai Cell Bank (Shanghai, China), cultured in DMEM supplemented with 4.5 g L^−1^ D-glucose, 10% FBS, and 1% penicillin at 37 °C containing 5% CO_2_. The cytotoxic effects of QAP and QWP on 3T3-L1 cells were measured following the previous method [15]. The 3T3-L1 adipocyte cells were seeded in a 96-well plate (5 × 10^4^ cells/well). After 18 h, the medium was replaced with DMEM containing 100 μL samples at varying concentrations. After 24 h, the culture medium was replaced with 100 μL of MTT solution (1 mg/mL). The plates were then incubated for an additional 4 h at 37 °C and 5% CO_2_. The MTT solution was removed, and 100 μL of dimethylsulfoxide (DMSO) was added for 30 min. The absorbance was measured at 570 nm. For the anti-proliferation assay, the 3T3-L1 adipocyte cells were cultivated in a 96-well plate (2.5 × 10^4^ cells/well). After 18 h, the medium was replaced with fresh DMEM containing 100 μL of samples at varying concentrations. Following a 72 h incubation at 37 °C in an environment with 5% CO_2_, the cells were subjected to staining with MTT for a duration of 30 min. Cell proliferation was quantified by measuring the absorbance at 570 nm using a multi-plate reader (Synergy H1, BioTek, Winooski, VT, USA).

### 2.4. Effect of Quinoa Polysaccharides on Transcriptional Level of 3T3-L1 Preadipocytes

The high-throughput sequencing platform (NovaSeq 6000, Illumina, Shanghai, China) was utilized for transcriptome sequencing [16]. FastQC (0.11.9) was used to analyze the data for quality control, and the high-quality sequences were compared with the reference genome in the Ensembl database to obtain the expression level of each gene [17]. Differentially expressed genes (DEGs) between untreated and quinoa polysaccharide-treated 3T3-L1 cell lines were identified. The condition was the differential expression multiple |log2foldchange| > 1, significance *p*-value < 0.05. Volcano and MA plots of DEGs were plotted using the ggplots2 software (3.4.1) package in R-Project. The distance was calculated using the Euclidean method, and the clustering was performed using the hierarchical clustering longest distance method. We mapped all genes that showed differential expression using the GO database. The term enriched by DEGs was calculated using hypergeometric distribution, with the whole genome as a reference. The count of DEGs at various levels of the Kyoto Encyclopedia of Genes and Genomes (KEGG) pathway was counted at various levels, and the primary metabolic and signaling pathways associated with these DEGs were identified. The pathways for significant enrichment of differential genes were calculated in the context of the whole genome. The hypergeometric distribution method was used to calculate the *p*-value (*p* < 0.05).

### 2.5. Quantitative RT-PCR (qPCR)

Q-PCR analysis was performed using total RNA extracted from 3T3-L1 preadipocytes that were cultured for 48 h. The reverse transcription process utilized random hexamer primers and was conducted using a reverse transcription system (TaKaRa, RR036A, Beijing, China). Gene-specific primers were generated using Primer Premier software (6.25), and GAPDH was employed as the internal reference gene for quantifying gene expression in qRT-PCR (Table 1). 

### 2.6. Data Statistics 

All data were measured in three replicates (means ± SD), and the analysis of variance (ANOVA) was followed by Duncan’s multiple-range test. The statistical analyses were conducted using SPSS version 22.0. Statistical significance was defined as *p* < 0.05.

## 3. Results and Discussion

### 3.1. Anti-Proliferation Effect of Quinoa Polysaccharides on 3T3-L1 Preadipocytes

**Figure 1:**
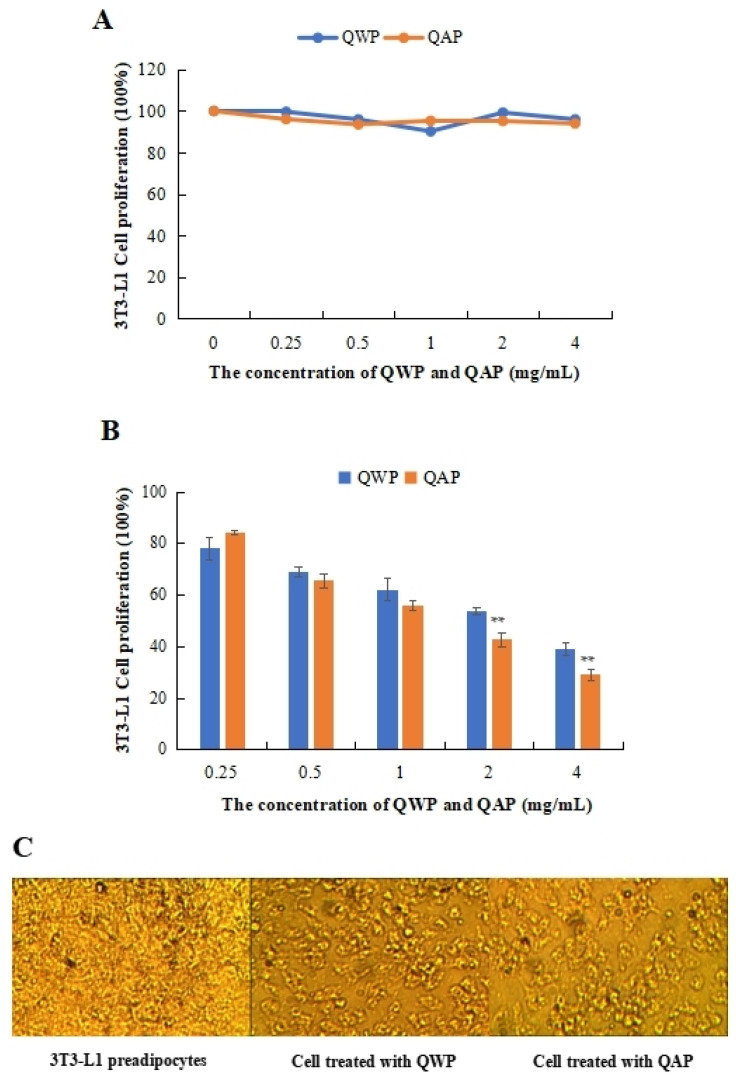
Anti-proliferative activity analysis of quinoa polysaccharides. (**A**) Cytotoxic effect of QWP and QAP on 3T3-L1 adipocytes. (**B**) QWP and QAP dramatically suppressed cell growth in the anti-proliferation experiment. (**C**) Topography of 3T3-L1 adipocytes and QWP- and QAP-treated samples. ** *p* < 0.01 shows significant differences. QAP represents quinoa alkaline-extracted polysaccharides. QWP represents quinoa water-extracted polysaccharides.

The cytotoxic and anti-proliferation effects of QWP and QAP on the 3T3-L1 preadipocytes were measured. QWP and QAP had no cytotoxic effects on 3T3-L1 preadipocytes within 0.25 to 4 mg/mL (Figure 1A). QAP showed stronger inhibitory activity than QWP at high concentrations, except at 0.25 mg/mL. At 4 mg/mL, QAP inhibited the proliferation of 3T3-L1 preadipocytes by 38.94%, which was higher than that of QWP (29.05%) (Figure 1B). In Figure 1C, after being treated with QWP and QAP, the number of 3T3-L1 preadipocytes was significantly reduced (Figure 1C).

In the 3T3-L1 preadipocyte differentiation experiment, treatment with quinoa polysaccharides could inhibit PPARγ, C/EBPα, C/EBPβ, and C/EBPδ [12]. In addition, previous studies found that quinoa polysaccharide showed an inhibition effect on the proliferation of SMMC 7721 cells [18]. However, the mechanism of inhibiting cell proliferation is not clear and is worthy of further study. Apoptosis is considered a kind of programmed cell death that can lead to characteristic changes, including blistering, cell contraction, nuclear fragmentation, chromatin condensation, and chromosome DNA fragmentation [19]. Cordyceps polysaccharide inhibits the expression of Cyclin E and CDK2, promotes cell apoptosis, and inhibits the proliferation of Hela cells [20]. Mushroom polysaccharides are rich in β-glucan, which can decrease the levels of lipid hydrogen peroxide in 3T3-L1 cells and effectively stimulate lipogenesis and lipolysis [21]. 

In order to further explore the global intracellular regulation of 3T3-L1 cells and reveal their anti-obesity function and possible molecular mechanisms, 3T3-L1 cells in the control group, 2 mg/mL, and 4 mg/mL QWP- and QAP-treated groups were collected and isolated for subsequent transcriptome analysis. Adipose tissue is an important endocrine organ in the body. It can secrete numerous adipokines to regulate energy metabolism, glucose and lipid metabolism, vascular homeostasis, and immune and reproductive processes in the body. As an important active ingredient, it regulates a variety of biological processes, including the regulation of blood pressure, immune responses, neurotransmitter transmission, and cell proliferation. We investigated the effects of two quinoa polysaccharides on the proliferation of 3T3-L1 preadipocytes, and the results showed that quinoa polysaccharides could inhibit the proliferation of 3T3-L1 preadipocytes, and QAP had a better inhibitory effect than QWP. The 3T3-L1 cell differentiation process is widely used in the study of adipocyte differentiation. However, there are few studies on the molecular mechanism of bioactive components affecting 3T3-L1 cells by transcriptomic technology. 3T3-L1 preadipocytes are widely used as cell models for studying obesity. Ginsenoside Rg1 could improve the phosphorylation level of AMP-activated protein kinase (AMPK) in 3T3-L1 cells to inhibit adipogenesis and reduce intracellular lipid content [22]. 

### 3.2. Analysis of Transcriptome Sequencing Results

**Figure 2:**
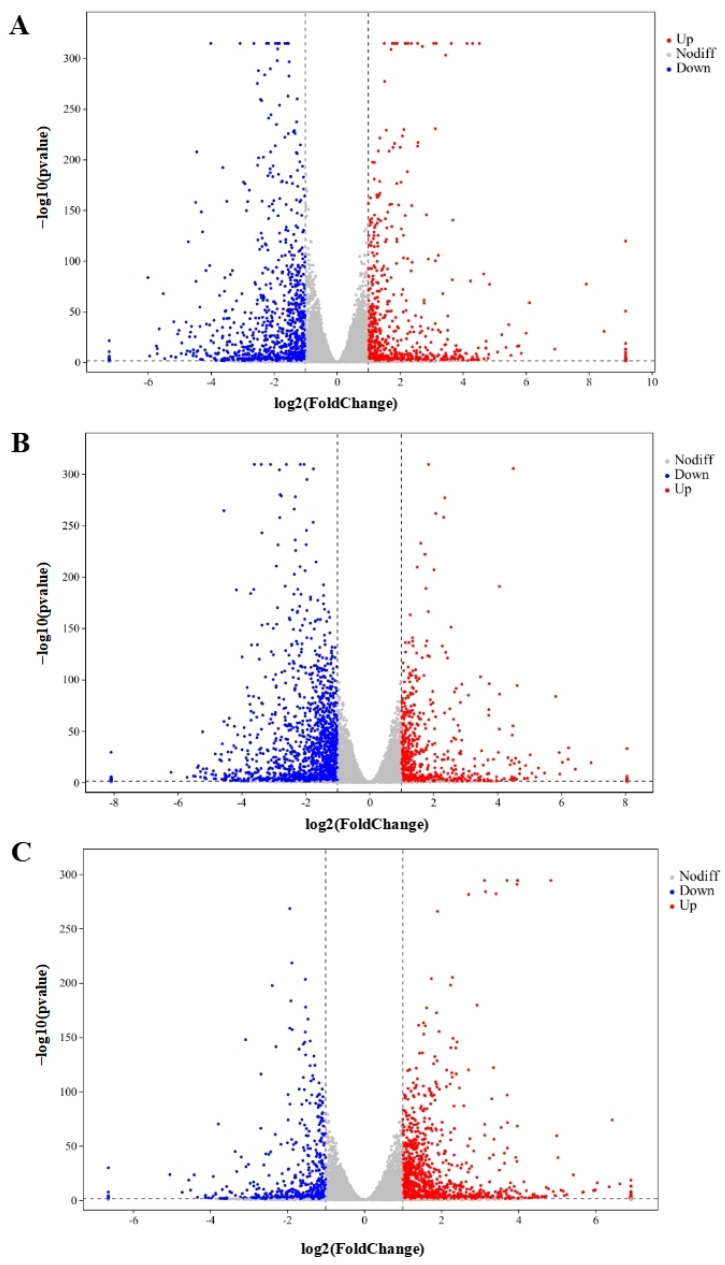
Genes that are expressed at variable levels between various treatment groups. The horizontal axis represents the logarithmic changes in gene expression among different groups. (**A**) CT vs. QAPHs; (**B**) CT vs. QWPHs; (**C**) QAPHs vs. QWPHs. The discrepancy was more pronounced when the adjusted *p*-value was less and the −log10 (adjusted *p*-value) was larger. The figure shows that the vertical bar represents a difference threshold that is 1.5 times higher, while the horizontal bar represents a threshold of statistical significance at *p* < 0.05. Distinct color splashes represented various genes, with gray dots indicating genes without notable variation, red dots indicating significantly upregulated genes, and blue dots relating to downregulated genes. CT represents the control group. QAPH represents high concentrations of quinoa alkaline-extracted polysaccharides. QWPH represents high concentrations of quinoa water-extracted polysaccharides.

**Figure 3:**
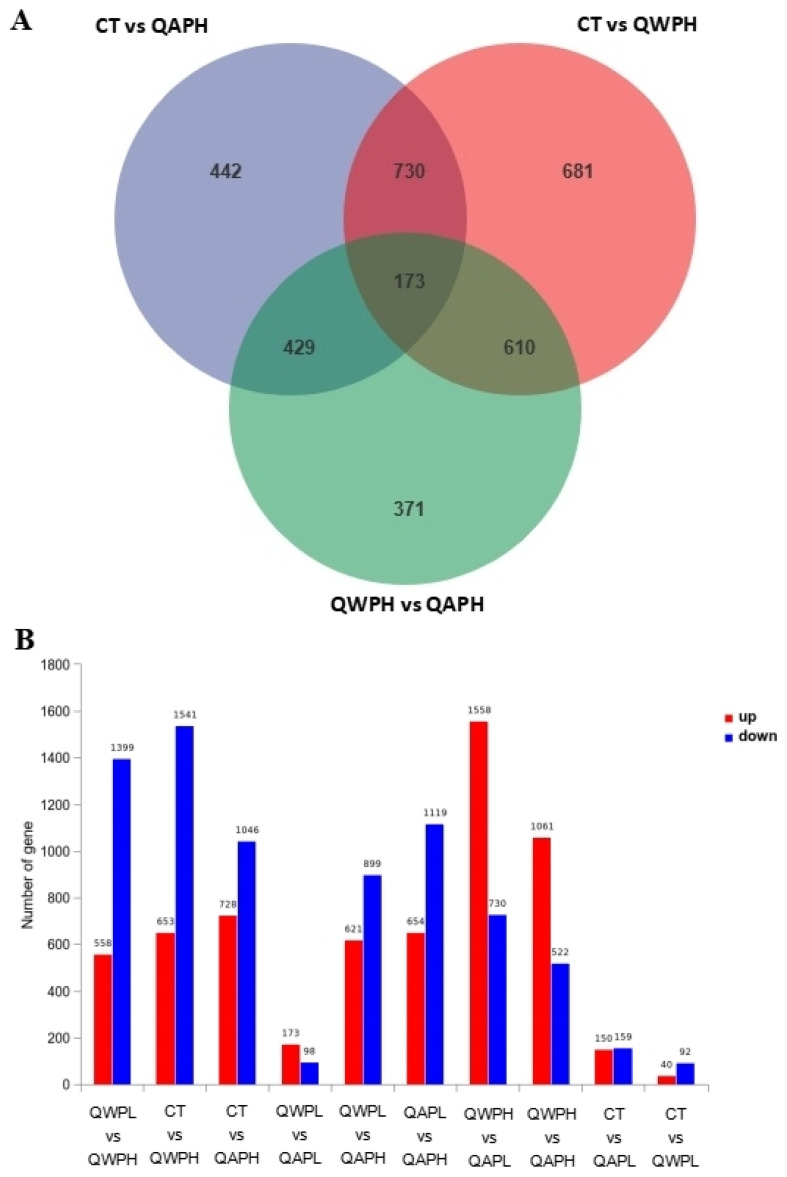
(**A**) Venn diagram illustrating the number of genes differing in overlap among comparison groups. (**B**) The upregulation and downregulation of genes.

**Figure 4:**
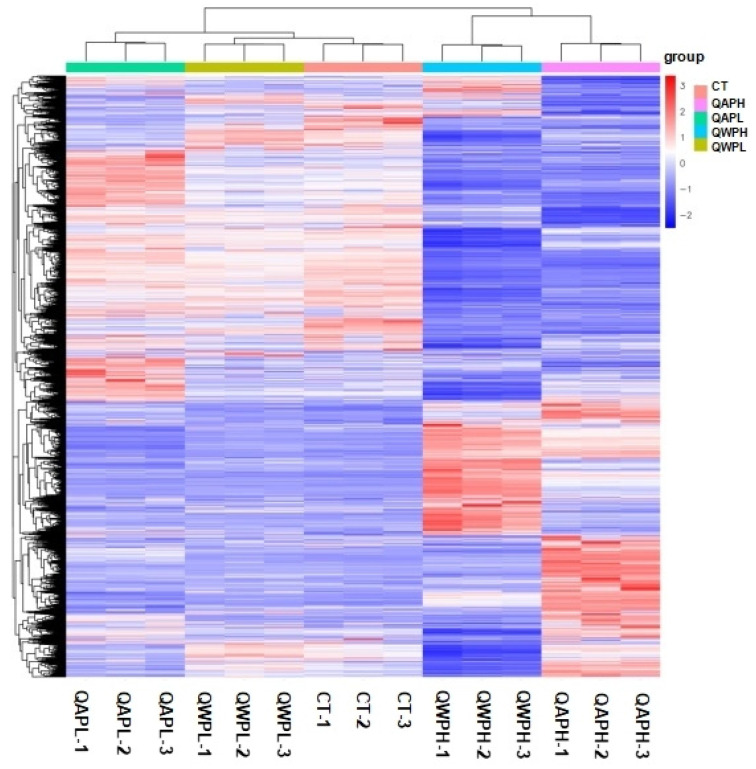
Heat map of mRNA transcripts showing hierarchical clustering of altered mRNA transcripts in 3T3-L1 preadipocytes with or without polysaccharide (QAP and QWP) treatment. Up- and downregulated genes are in red and blue, respectively. QAP represents quinoa alkaline-extracted polysaccharides. QWP represents quinoa water-extracted polysaccharides.

In total, 48,878,089, 49,409,051, 55,533,208, 51,156,189, and 53,245,847 clean reads were recognized from QAPHs, QAPLs, QWPHs, QWPLs, and the control group (CT), respectively. The extracted total RNA is shown in Figure S1. The electrophoresis results of all RNA samples showed two bands of 28S and 18S. In addition, the values of RIN and 28S/18S in all RNA samples were more than 9.8 and 1.6 (Table S1). All these results showed that the RNA extracted in this experiment is of high quality and can be used in subsequent experiments. The mapping ratios were both over 95.8%, respectively, showing significant levels of gene expression (Table S2). The base content distribution is generally used to determine the presence of separation between A and T, G and C. And it is basically stable throughout the entire sequencing process, showing a horizontal line. The use of 6 bp random primers during reverse transcription synthesis of cDNA can lead to normal variations that result in a specific preference for the nucleotide composition of the initial locations (Figure S2). Between CT and QWPHs, 2194 genes were significantly expressed, including 653 upregulated and 1541 downregulated. Between CT and QAPHs, there were 1774 DEGs, including 728 upregulated and 1046 downregulated (Figure 2, Figure 3 and Figure 4). The number of DEGs was determined by summing the number of genes in each colored circle. The Venn plot illustrates the common DEGs between the two control groups (Figure 3A).

There were three levels in the expression analysis of the transcriptome, including gene, transcript, and exon expression levels [23]. Diverse splicing variants of a single gene can give rise to distinct biological consequences. The FPKM density distribution offers a comprehensive examination of the expression patterns of all genes in the sample. Most genes exhibit moderate expression levels, with a smaller fraction being expressed at either a low or high level. Our results correspond to these descriptions (Figure S3). 

In this study, the correlation coefficients between the biologically replicated samples are higher than 0.9, indicating that the correlation between the samples is high (Figure S4). In addition, PCA analysis shows high similarity between samples. Recently, transcriptome sequencing technology has developed rapidly and is widely used to identify cell engineering. Designing a transcriptome-guided cell engineering system requires a large number of samples to accurately predict key genes. 

### 3.3. Differential Expression Analysis 

Obesity is a complex biological process. A better understanding of various signaling molecules will help elucidate the mechanisms that inhibit the proliferation and development of 3T3-L1 cells. Previous transcriptome sequencing showed that Isocitrate dehydrogenase 2 (Idh2), fatty acid synthase (Fasn), Lpin1, and lipoproteinlipase (Lpl) were affected by all-trans retinoic acid and made to undergo significant changes, indicating that these genes may be inhibiting the differentiation of 3T3-L1 cells and play a key role [24]. In this study, ten DEGs were identified after QAP and QWP treatments compared with the blank group. Fold changes of ATPase 13A4 (ATP13A4) between CT and QAPLs, CT and QAPHs were 7.231 and 574.614. It is worth noting that ATP13A4 expression decreased after treatment with QWP. Fold changes of ATPase 13A4 (ATP13A4) between CT and QAPLs, CT and QAPHs were 3.579 and 65.734. ATP13A4 is a cation-transporting P5-type ATPase that is linked to neurodevelopmental disorders. Studies have shown that the loss of ATP13A4 is related to neuropsychiatric phenotypes [25]. In this study, after quinoa polysaccharide treatment, the expression of ATP13A4 increased, indicating that QWP and QAP can inhibit the symptoms of obesity caused by neurodevelopmental disorders. The Globoside alpha-1,3-N-acetylgalactosaminyltransferase 1 (Gbgt1) gene is involved in encoding forssman glycosphingolipid synthase, and its presence leads to the expression of FORS1 glycosphingolipids in human red blood cells [26]. The Gbgt1 expression of 3T3-L1 cells treated with QAP and QWP was lower than that in the untreated group. Fold changes of GBGT1 between CT and QAPLs, CT and QAPHs were 0.92 and 0.06. Fold changes of GBGT1 between CT and QWPLs, CT and QWPHs were 0.604 and 0.024. Moreover, the expression of GBGT1 was more inhibited after high-concentration quinoa polysaccharide treatment than after low-concentration quinoa polysaccharide treatment (Table 2 and Table 3). 

### 3.4. GO Analysis of DEGs

**Figure 5:**
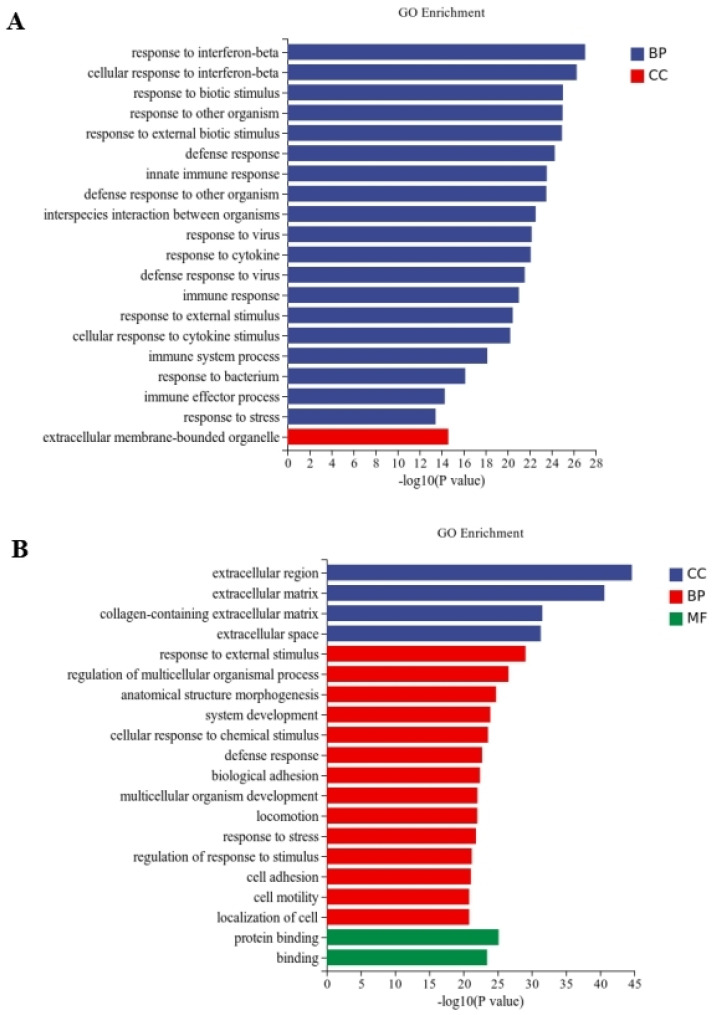
Histogram difference genes’ GO enrichment between CT and QAP treatment. The abscissa is −log10 (*p*-value) enriched by GO Term, and the ordinate is GO Term. CC stands for cellular component, BP stands for biological process, and MF stands for molecular function. (**A**) CT vs. QAPLs; (**B**) CT vs. QAPHs. CT represents control group. QAPL represents low-concentration quinoa alkaline-extracted polysaccharide. QAPH represents high-concentration quinoa alkaline-extracted polysaccharide.

**Figure 6:**
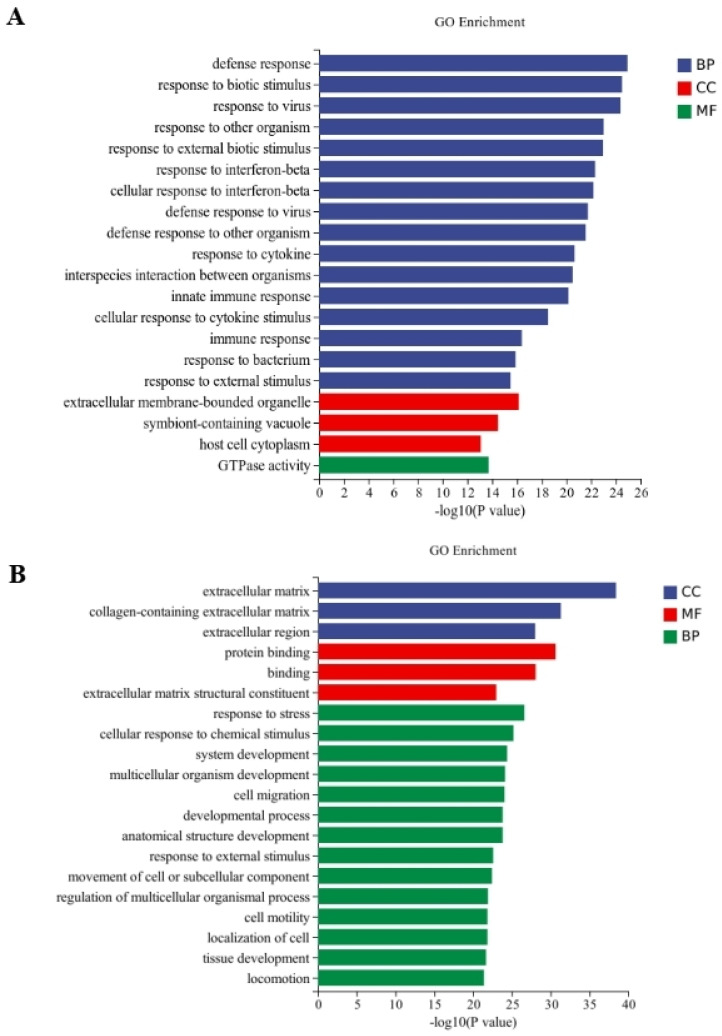
Histogram difference genes’ GO enrichment between CT and QWP treatment. The abscissa is −log10 (*p*-value) enriched by GO Term, and the ordinate is GO Term. CC stands for cellular component, BP stands for biological process, and MF stands for molecular function. (**A**) CT vs. QWPLs; (**B**) CT vs. QWPHs. CT represents control group. QWPL represents low-concentration quinoa water-extracted polysaccharide. QWPH represents high-concentration quinoa water-extracted polysaccharide.

Between the CT and QAPL treatment groups, nineteen categories and one category were included in the biological process and cellular component, respectively, in which the response to the interferon-beta pathway showed the most significant difference (Figure 5A). The comparison between the CT and QAPH treatment groups revealed that there were four categories included in the cellular component, fourteen categories included in the biological process, and two categories included in the molecular function. Among these categories, the extracellular region exhibited the most significant difference (Figure 5B). Between the CT and QWPL treatment groups, sixteen categories, three categories, and one category were included in biological processes, cellular components, and molecular function. Defense response was the most significant pathway (Figure 6A). Between the CT and QWPH treatment groups, three, three, and fourteen categories were included in the cellular component, molecular function, and biological process. It was found that the extracellular matrix was the most significant change pathway (Figure 6B). The GO enrichment analysis of DEGs revealed that the cellular process in 3T3-L1 cells was the function most significantly affected by quinoa polysaccharides.

### 3.5. KEGG Pathway Analysis of DEGs

Pathway significance enrichment analysis identified the involvement of many genes in biochemical metabolic and signal transduction pathways. To further understand the biological functions involved in the differential genes, KEGG analysis was performed on these 2194 differential genes.

**Figure 7:**
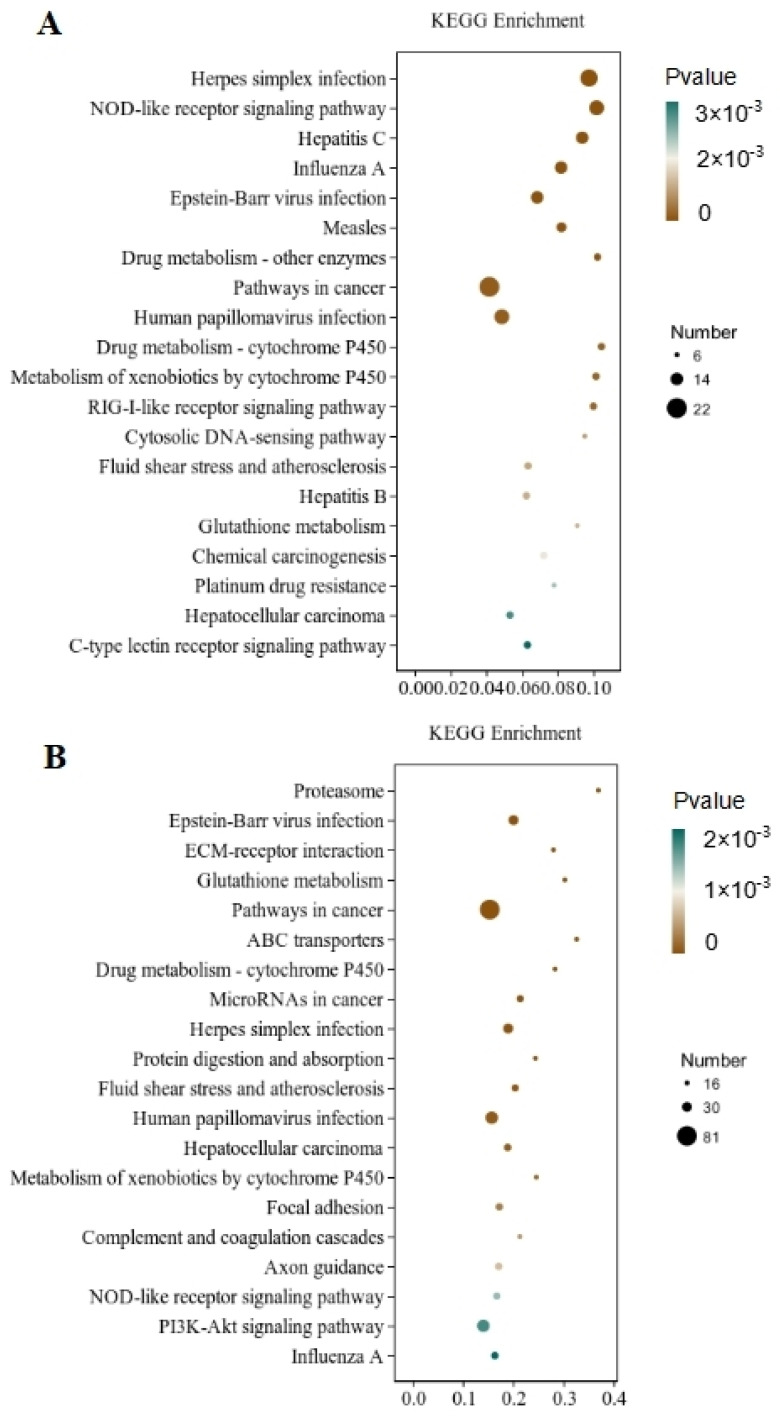
KEGG pathway rich enrichment. (**A**) CT vs. QAPLs; (**B**) CT vs. QAPHs. CT represents the control group. QAPL represents low-concentration quinoa alkaline-extracted polysaccharide. QAPH represents high-concentration quinoa alkaline-extracted polysaccharide.

**Figure 8:**
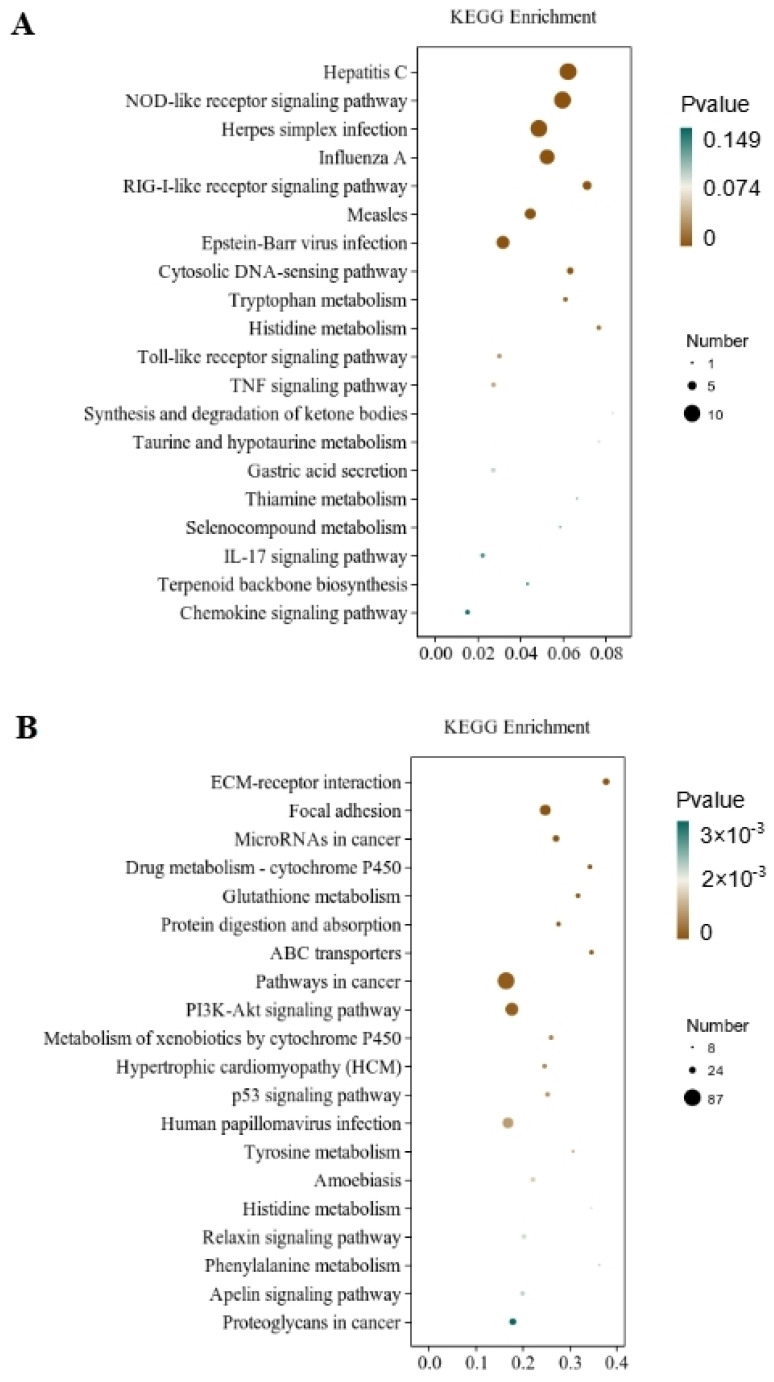
KEGG pathway rich enrichment. (**A**) CT vs. QWPLs; (**B**) CT vs. QWPHs. CT represents the control group. QWPL represents low-concentration quinoa water-extracted polysaccharide. QWPH represents high-concentration quinoa water-extracted polysaccharide.

Through the significant enrichment analysis of the pathway, the metabolic and signal transduction pathways involved in differential gene involvement were identified. There were 212 metabolic pathways with significant expression differences identified in the CT and QAPLs groups, 35 of which showed the most significant differences (*p* < 0.05, Figure 7A). A total of 302 metabolic pathways with significant expression differences were identified between the CT and QAPHs, 35 of which showed the most significant differences (*p* < 0.05, Figure 7B). A total of 84 metabolic pathways with significant expression differences were identified between the CT and QWPLs, 12 of which showed the most significant differences (*p* < 0.05, Figure 8A). A total of 310 metabolic pathways with significant expression differences were identified between the CT and QWPHs, 57 of which showed the most significant differences (*p* < 0.05, Figure 8B). Significantly enriched pathways included Herpes simplex infection, NOD-like receptor signaling pathway, Hepatitis C, Influenza A, and PI3K-Akt signaling pathway, which is associated with human diseases, immunology pathways metabolism, genetic information processing, immunity, apoptosis, proliferation, and adhesion. The compound has the potential to trigger programmed cell death in 3T3-L1 preadipocytes by blocking the activity of nuclear factor kappa-B (NF-κB) and modifying the mitogen-activated protein kinases (MAPKs) pathway [11]. The effect of tuckahoe flavonoids was determined on 3T3-L1 cells by transcriptome technology, and the sample had no cytotoxic effect at a concentration of 250 μg/mL [14]. A total of 1529 genes (657 upregulated and 872 downregulated) were significantly differentially expressed after cocos flavonoids treatment. The GO enrichment analysis revealed a significant enrichment in extracellular matrix tissue, aging, and inflammatory responses between blank and sample tissues. It was found that the key pathways of all genes mostly include the lysosome, focal adhesion, TGF-β signaling pathway, calcium signaling route, ECM-receptor interaction, and PI3K-Akt signaling pathway, as identified using KEGG analysis. This result is consistent with this study, which strongly confirms the mechanism of the effects of quinoa polysaccharide components QWP and QAP on 3T3-L1 cells, laying a foundation for the potential therapeutic utility in the prevention of obesity and associated metabolic disorders. The PI3K/AKT pathway contributes to regulating cell proliferation [27]. NOD-like receptors (NLRs) are recognized as essential regulators in disorders related to inflammation [28]. In CT vs. QAPHs, the proteasome pathway is significantly enriched, which is related to the pathogenesis of many diseases. Cell proteolysis is carried out by a complex cascade of enzymes and shows a high degree of specificity for many of its substrates. This study demonstrates that polysaccharides derived from quinoa have the ability to hinder the growth and cell division of 3T3-L1 cells by inhibiting the PI3K/AKT pathway and the NOD-like receptor signaling pathway.

### 3.6. Structural Analysis

At the genetic level, sequencing reads can be conducted by comparing the reference genome for expression analysis, expression difference analysis, and enrichment analysis, and a series of structural analyses can also be performed on the transcript level. We compare the spliced transcript sequence with known transcripts to obtain transcripts without annotation information. As shown in Figure S5, 91.7% belong to new potential transcripts with at least one alternative splicing site shared with known transcripts. The transcript fragment is completely in a known intron, which accounts for 2.8% and 2.1%, respectively. Exons are overlaid on the anti-strand of a known gene, and 3.3% of them belong to an unknown fragment.

### 3.7. Transcription Factor Analysis

Transcription Factor (TF) is a protein molecule that has the ability to selectively attach to a certain sequence located upstream of the 5′ end of a gene. It forms a complex with RNA polymerase II, which initiates the process of transcription [29]. In this study, the DEGs predicted to be transcription factors were counted. According to the family information, Figure S6 shows the number of differentially expressed transcription factors in the blank group and each sample group. The results indicated that the Zf-C2H2 transcription factor family had the largest number of enriched differential transcription factors. In the comparison of CT and QWPL treatment groups, 12 upregulated and 23 downregulated transcription factors were identified. The Zf-C2H2 transcription factor family has the most enriched differential transcription factors between CH and QWPH treatment groups, including seven upregulated and nineteen downregulated transcription factors. Zinc finger (Zf) is a compact protein structure that is defined by the binding of one or more zinc ions (Zn^2+^) to promote folding and contains a variety of different protein structures. Despite the wide range of these proteins, the vast majority typically serve as interacting modules that bind DNA, RNA, proteins, or other useful small molecules, and changes in structure are primarily employed to modify the binding specificity of a particular protein.

This study explored the regulatory mechanisms of QWP and QAP inhibiting proliferation of 3T3-L1 preadipocytes. Metabolomics technology should be used to find the intracellular differential metabolites caused by the action of quinoa polysaccharides and to explore the relevant metabolic pathways in the process of differentiation and proliferation.

### 3.8. Quantitative RT-PCR (qPCR) Validation

**Figure 9:**
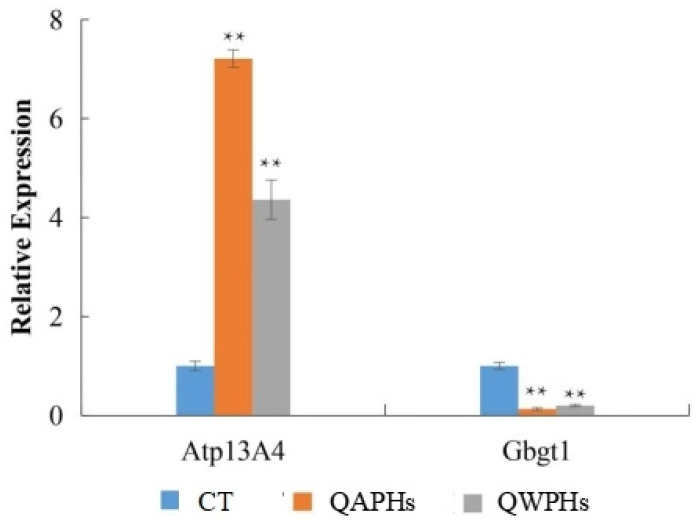
qPCR validation of DEGs. Mean ± SD. *n* = 3. ** *p* < 0.01 vs. CT group. CT represents the control group. QAPH represents the high-concentration quinoa alkaline-extracted polysaccharide. QWPH represents the high-concentration quinoa water-extracted polysaccharide. DEG represents differentially expressed gene.

To validate the findings of transcriptome sequencing and conduct a more detailed analysis of gene expression patterns related to anti-obesity functions, Atp13A4 and Gbgt1 were selected for quantitative RT-PCR due to their distinct expression patterns. The RT-PCR results demonstrated that the expressions of Atp13A4 were significantly higher (*p* < 0.05) in 3T3-L1 preadipocytes treated with QAPHs and QWPHs compared to the control group. Conversely, the expression of Gbgt1 was significantly reduced (*p* < 0.05, Figure 9) in the treated group.

## 4. Conclusions

To summarize, the quinoa polysaccharide has a notable ability to prevent the growth of 3T3-L1 preadipocytes in vitro. This impact is achieved through the interplay of many pathways and signaling molecules. Interference with many signaling pathways, such as NOD-like receptor signaling pathway, Herpes simplex infection, Hepatitis C, PI3K-Akt signaling pathway, and many factors, such as Atp13A4 and Gbgt1, affect the proliferation and apoptosis of 3T3-L1 preadipocytes. Subsequently, the differential lipid metabolites produced by quinoa polysaccharides on 3T3-L1 preadipocytes could be analyzed in conjunction with lipidomics to explore their anti-obesity effects. This study established a solid theoretical basis for investigating the anti-obesity mechanisms of QWP and QAP. In addition, quinoa polysaccharides can be further used as a kind of anti-obesity agent.

## Figures and Tables

**Table 1:** Primers and sequences used in qRT-PCR analysis.

Gene	Primers	Sequences (5′→3′)
Atp13A4	Atp13A4-F	CACGTATGGGCACATTGTGTC
	Atp13A4-R	TGAGACCAAATGCGCTGTTTA
Gbgt1	Gbgt1-F	TGGGTGTATCTTGAGAACTGGC
	Gbgt1-R	GTACTGTGACCATACCACGGG
GAPDH	GAPDH-F	GGGAGCCAAAAGGGTCATCA
	GAPDH-R	TGATGGCATGGACTGTGGTC

**Table 2:** Fold changes of DEG treated with QAP.

Gene id	Name	CT vs. QAPLs	CT vs. QAPHs	QAPLs vs. QAPHs
ENSMUSG00000038094	ATP13A4	7.231	574.614	79.74
ENSMUSG00000051228	Nyx	2.656	69.157	26.1
ENSMUSG00000057933	Gsta2	1.478	37.748	25.636
ENSMUSG00000021062	Rab15	1.697	24.484	24.484
ENSMUSG00000111709	Gm3776	1.176	28.313	24.15
ENSMUSG00000018656	Tcaf3	1.02	22.454	22.121
ENSMUSG00000026012	Cd28	1.026	20.365	19.944
ENSMUSG00000063851	Rnf183	12.741	242.082	19.058
ENSMUSG00000038963	Slco4a1	1.429	25.326	17.766
ENSMUSG00000056457	Prl2c3	1.02	17.553	17.26
ENSMUSG00000041559	SLRR2E	0.739	0.019	0.026
ENSMUSG00000020676	Ccl11	0.86	0.033	0.038
ENSMUSG00000027220	Syt13	0.798	0.036	0.045
ENSMUSG00000040170	Fmo2	0.611	0.029	0.048
ENSMUSG00000037206	Islr	0.862	0.046	0.053
ENSMUSG00000078922	Tgtp1	0.347	0.019	0.667
ENSMUSG00000042436	Mfap4	0.813	0.047	0.058
ENSMUSG00000026829	Gbgt1	0.92	0.06	0.06
ENSMUSG00000020053	Igf1	0.734	0.045	0.061
ENSMUSG00000034009	Rxfp1	0.264	0.016	0.062

**Table 3:** Fold changes of DEG treated with QWP.

id	Name	CT vs. QWPLs	CT vs. QWPHs	QWPLs vs. QWPHs
ENSMUSG00000059383	Gfral	1.733	75.096	43.574
ENSMUSG00000020646	Mboat2	2.039	74.532	36.663
ENSMUSG00000038094	Atp13A4	3.579	65.734	18.422
ENSMUSG00000039691	Tspan10	3.079	62.112	20.235
ENSMUSG00000030827	Fgf21	6.198	61.422	9.954
ENSMUSG00000029797	Sspo	2.387	56.769	23.88
ENSMUSG00000040026	Saa3	7.253	44.579	6.188
ENSMUSG00000026822	Lcn2	2.594	39.139	15.152
ENSMUSG00000009356	Lpo	3.028	37.007	12.247
ENSMUSG00000034402	Kcnh5	2.638	33.928	12.916
ENSMUSG00000031364	Grpr	0.679	0.004	0.005
ENSMUSG00000022468	Endou	0.855	0.019	0.023
ENSMUSG00000024810	Il33	0.85	0.024	0.029
ENSMUSG00000027238	Frmd5	0.908	0.028	0.031
ENSMUSG00000048368	Omd	0.806	0.029	0.036
ENSMUSG00000031554	Adam5	0.956	0.036	0.037
ENSMUSG00000085584	Rtl9	0.884	0.033	0.038
ENSMUSG00000024059	Clip4	0.889	0.034	0.038
ENSMUSG00000026829	Gbgt1	0.604	0.024	0.04
ENSMUSG00000048782	Insc	0.538	0.022	0.041

## Data Availability

The original contributions presented in the study are included in the article/Supplementary Materials, further inquiries can be directed to the corresponding authors.

## Supplementary Materials

The following supporting information can be downloaded at: https://www.mdpi.com/article/10.3390/foods13152311/s1, Figure S1: Agarose gel electrophoresis of total RNA, M: Marker, 1–3: CT, 4–6: QWPLs, 7–9: QWPHs, 10–12: QAPLs, 13–15: QAPHs; Figure S2: Distribution of basic group. The abscissa shows the base position in Reads (5’–>3’). The ordinate shows the proportion of bases. (a) CT, (b) QAPLs, (c) QAPHs, (d) QWPLs, (e) QWPHs; Figure S3: (a) The abscissa shows the log10 (FPKM) value of the gene, and the ordinate shows the gene distribution density corresponding to the expression level, (b) PCA analysis between samples. Different colors represent different groups; Figure S4: correlation of gene expression levels between samples; Figure S5: New transcript analysis. (a) New Transcript Class Code Distribution (i: the transcript fragment is completely in a known intron, j: new potential transcripts: at least one alternative splicing site shared with known transcripts, x: Exons are overlaid on the anti-strand of a known gene, u: unknown fragment, transcript in the intergenic region). (b) Sequence number of different databases; Figure S6: (a) Analysis of transcription factors; Differentially expressed transcription factor distribution, (b) CT vs. QAPLs, (c) CT vs. QAPHs, (d) CT vs. QWPLs, (e) CT vs. QWPHs; Table S1: The values of RIN and 28S/18S in all RNA samples.; Table S2: Quality of sequencing data.
