# Supplementary material for: Transcriptome Analysis Reveals the Mechanism of Quinoa Polysaccharides Inhibiting 3T3-L1 Preadipocyte Proliferation
Source: Foods. 2024 Jul 23;13(15):2311. doi: 10.3390/foods13152311 (PMC11311824; doi:10.3390/foods13152311)
Supplement: Supplementary file 1 [file foods-13-02311-s001.zip › foods-3039091-supplementary.pdf]

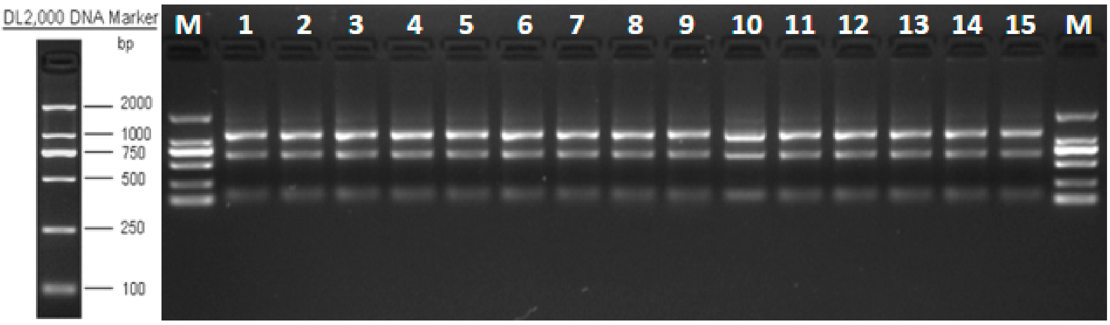

Figure S1. Agarose gel electrophoresis of total RNA, M: Marker, 1-3: CT, 4-6: QWPLs, 7-9: QWPHs, 10-12: QAPLs, 13-15: QAPHs.

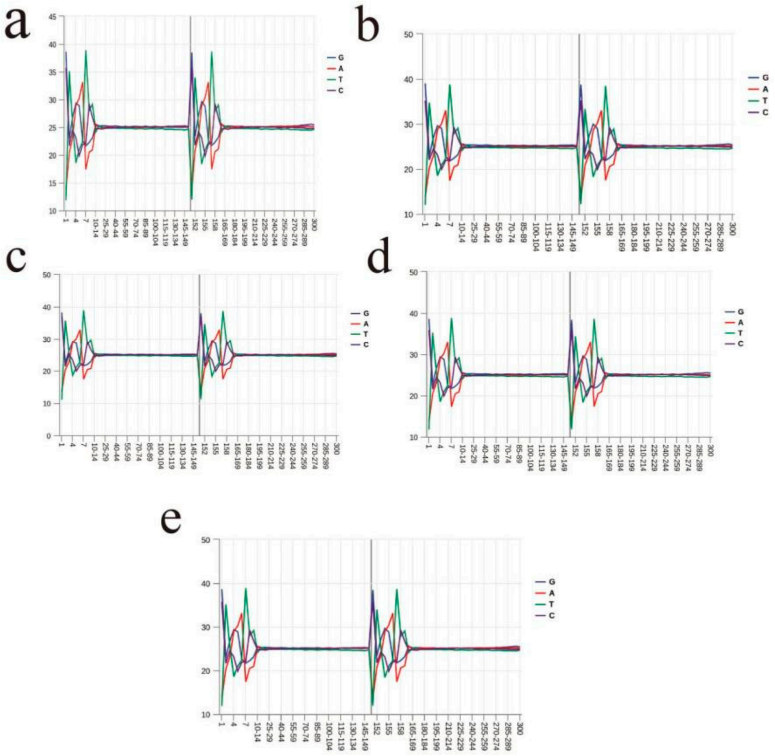

Figure S2. Distribution of basic group. The abscissa shows the base position in Reads (5'->3'). The ordinate shows the proportion of bases. (**a**) CT, (**b**) QAPLs, (**c**) QAPHs, (**d**) QWPLs, (**e**) QWPHs.

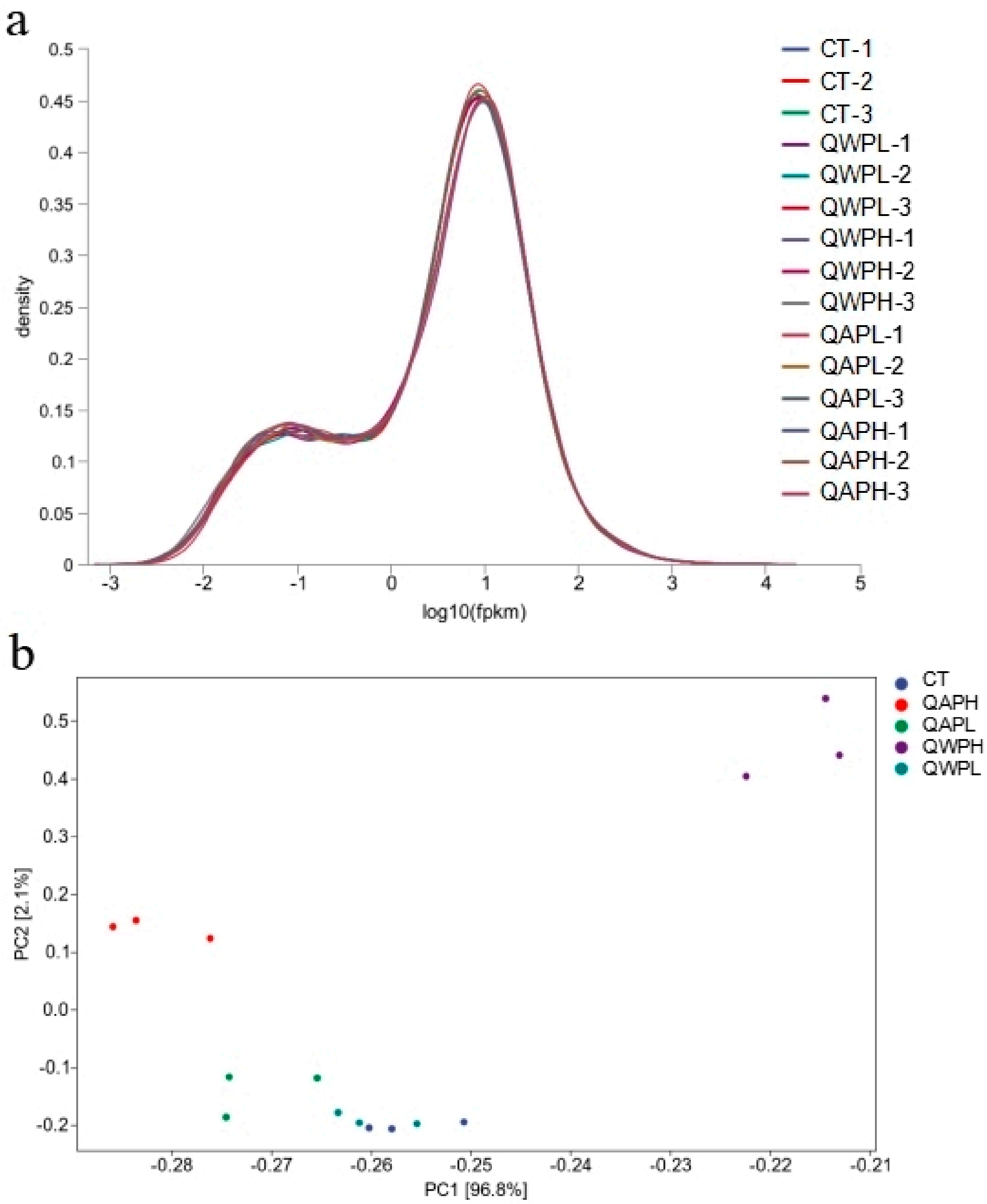

Figure S3. (**a**) The abscissa shows the log10 (FPKM) value of the gene, and the ordinate shows the gene distribution density corresponding to the expression level, (**b**) PCA analysis between samples. Different colors represent different groups.

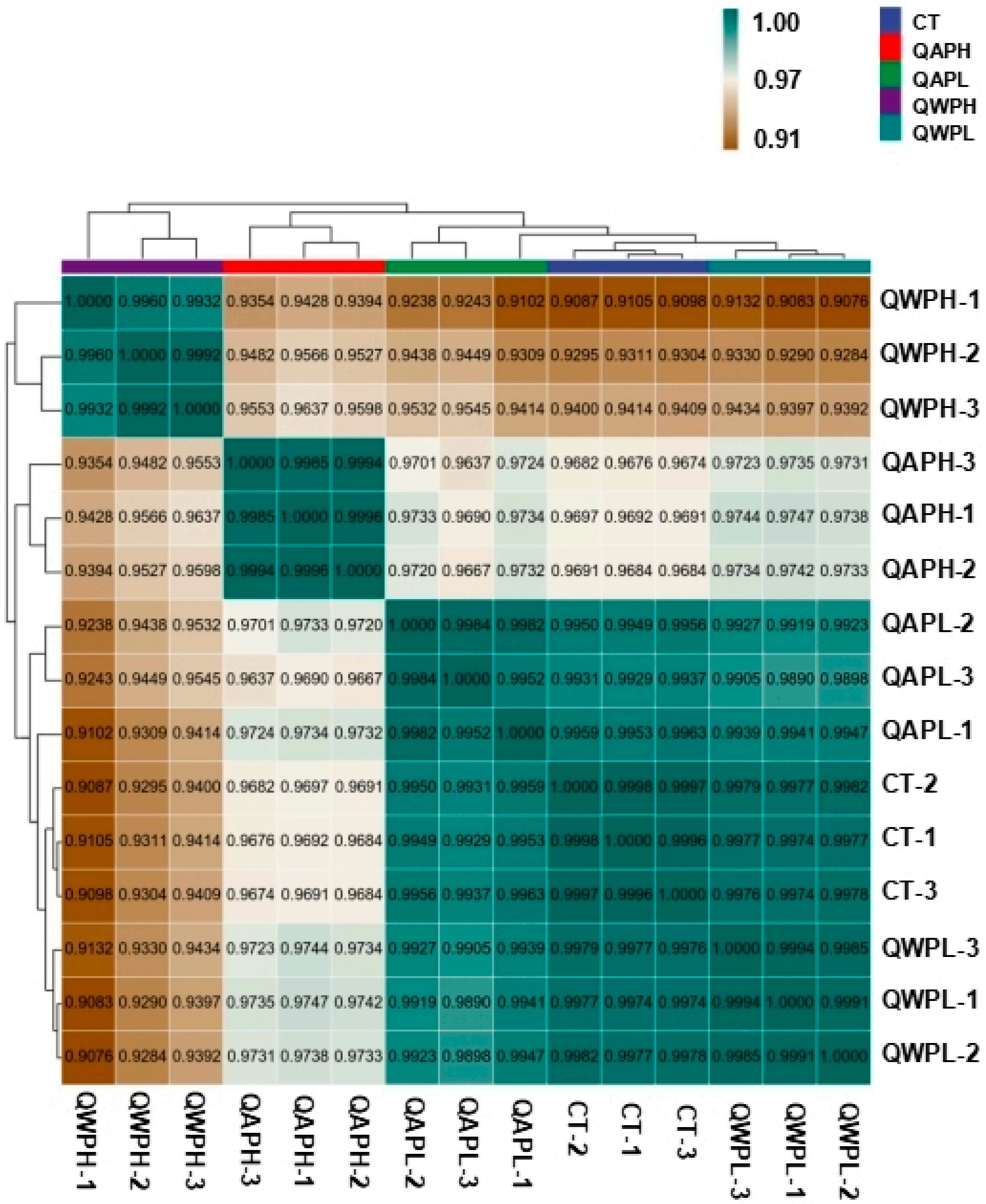

Figure S4. Correlation of gene expression levels between samples.

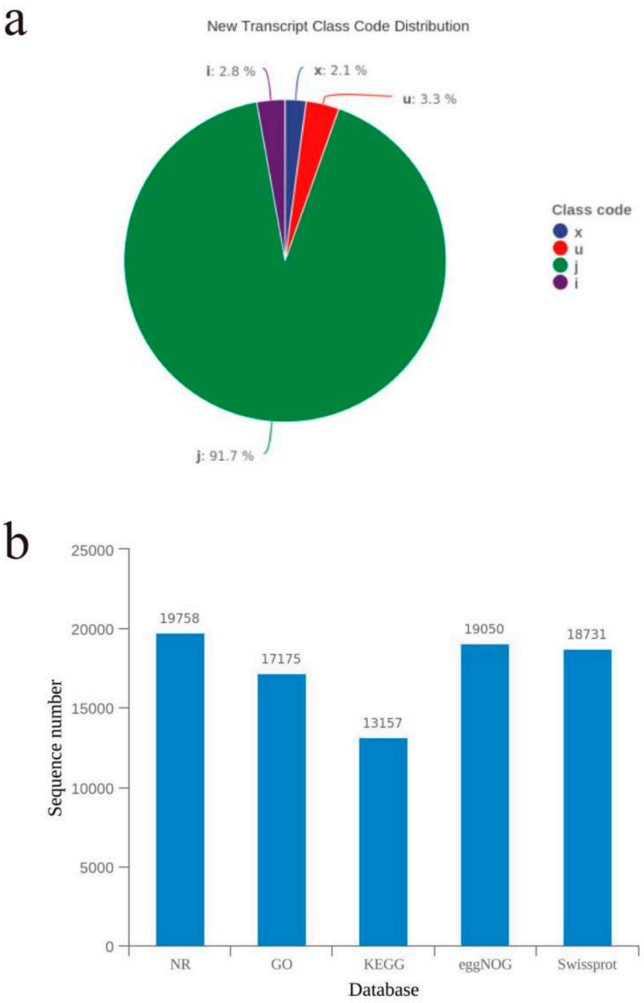

Figure S5. New transcript analysis. (**a**) New Transcript Class Code Distribution (i: the transcript fragment is completely in a known intron, j: new potential transcripts: at least one alternative splicing site shared with known transcripts, x: Exons are overlaid on the anti-strand of a known gene, u: unknown fragment, transcript in the intergenic region). (**b**) Sequence number of different databases.

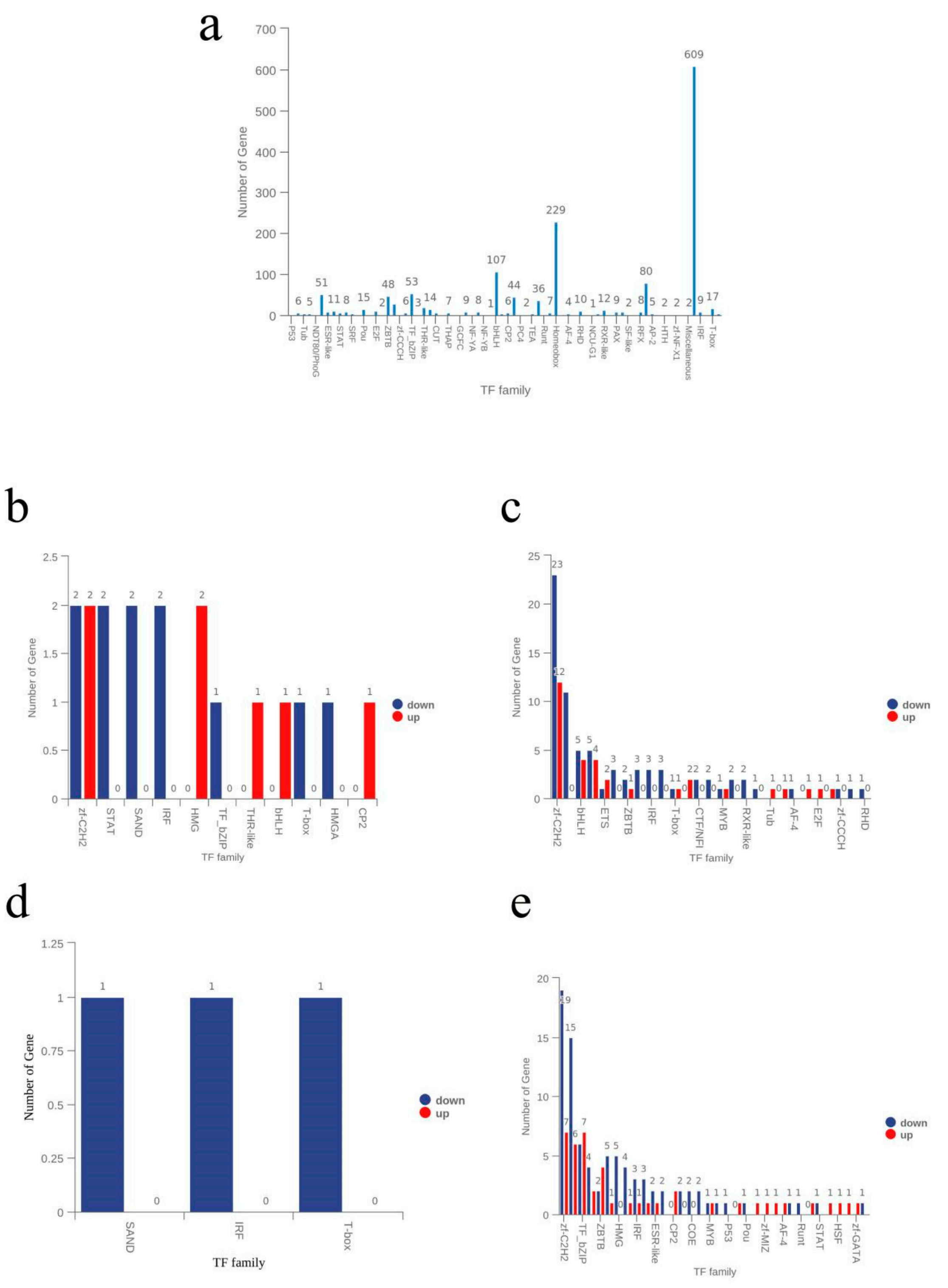

Figure S6. (**a**) Analysis of transcription factors; Differentially expressed transcription factor distribution, (**b**) CT vs QAPLs, (**c**) CT vs QAPHs, (**d**) CT vs QWPLs, (**e**) CT vs QWPHs.

Table S1. The values of RIN and 28S/18S in all RNA samples

Sample	RIN value	28S/18S
CT1	10	1.6
CT2	10	1.7
CT3	10	1.6
QAPHs1	9.9	1.7
QAPHs2	10	1.7
QAPHs3	10	1.7
QAPLs1	9.8	1.8
QAPLs2	9.8	1.8
QAPLs3	9.8	1.8
QWPHs1	10	1.7
QWPHs2	10	1.7
QWPHs3	10	1.7
QWPLs1	10	1.6
QWPLs2	10	1.6
QWPLs3	10	1.6

Table S2. Quality of sequencing data

Sample	Clean_Reads	Total_Mapped	Uniquely_Mapped
CT1	49,480,764	47,451,638 (95.90%)	45,007,373 (94.85%)
CT2	49,905,990	47,837,356 (95.85%)	45,323,694 (94.75%)
CT3	49,128,360	47,184,955 (96.04%)	44,714,151 (94.76%)
QAPHs1	45,318,002	43,505,284 (96.00%)	41,132,900 (94.55%)
QAPHs2	46,525,238	44,607,093 (95.88%)	42,181,265 (94.56%)
QAPHs3	44,025,824	42,200,811 (95.85%)	39,986,966 (94.75%)
QAPLs1	41,486,986	39,753,537 (95.82%)	37,709,717 (94.86%)
QAPLs2	47,409,214	45,457,879 (95.88%)	43,132,885 (94.89%)
QAPLs3	48,726,168	46,714,926 (95.87%)	44,189,827 (94.59%)
QWPHs1	51,975,082	49,951,227 (96.11%)	47,706,294 (95.51%)
QWPHs2	51,299,318	49,224,149 (95.95%)	46,998,432 (95.48%)
QWPHs3	52,125,082	49,959,338 (95.85%)	47,623,305 (95.32%)
QWPLs1	47,286,994	45,403,494 (96.02%)	42,989,611 (94.68%)
QWPLs2	46,404,190	44,495,334 (95.89%)	42,168,684 (94.77%)
QWPLs3	48,655,996	46,771,869 (96.13%)	44,273,861 (94.66%)
